# Home and Community Features, Perceived Age-Friendliness, and Intention Toward Aging in Place

**DOI:** 10.1093/geroni/igab046.2142

**Published:** 2021-12-17

**Authors:** Yeon Jin Choi

**Affiliations:** University of Southern California, Los Angeles, California, United States

## Abstract

Promoting age-friendliness of communities and supporting aging in place (AIP) are of great importance in aging societies. However, little is known about the mechanism linking home and neighborhood features, older adults’ global assessment of community, and their willingness to age-in-place despite the importance in developing policies and interventions. This study used the 2015 AARP Age-Friendly Community Survey, which includes 66 home and neighborhood features under the eight domains specified by the WHO’s Age-Friendly Cities Guidelines. A series of linear regression models were estimated to examine the interrelationship between the availability of age-friendly features in eight domains, perceived age-friendliness of community, and intention toward AIP. Overall, a greater availability of age-friendly features was positively associated with perceived age-friendliness of community and AIP intention. The relationship between age-friendly features and AIP intention was mediated by perceived age-friendliness of community (50.3% to 96% of the total effects). When perceived age-friendliness of community was introduced to models, the direct effects of housing, outdoor spaces and buildings, and transportation domains remained significant. Findings suggest that a greater availability of age-friendly features influence older adults’ perception on their community, leading to the development of a desire to age-in-place. Domains of housing, outdoor spaces and buildings, and transportation may be the most importance features in promoting age-friendliness of community and the key determinants of aging-in-place. Policy makers and practitioners may need to prioritize promoting age-friendly built environment before social environment in building age-friendly communities.

